# Research Progress in the Evaluation of Thermal Shock Resistance of Refractories: From Theoretical Evolution to Intelligent Characterization

**DOI:** 10.3390/ma19112337

**Published:** 2026-06-01

**Authors:** Gang Wang, Bo Ren, Jingjing Liu, Enhui Wang, Xinmei Hou, Mao Chen

**Affiliations:** 1 State Key Laboratory of Comprehensive Utilization of Vanadium and Titanium Resources, Panzhihua 617000, China; 2 School of Materials Science and Engineering, University of Science and Technology Beijing, Beijing 100083, China; 3 Technical Support Center for Prevention and Control of Disastrous Accidents in Metal Smelting, University of Science and Technology Beijing, Beijing 100083, China; 4 Institute for Carbon Neutrality, University of Science and Technology Beijing, Beijing 100083, China

**Keywords:** refractories, thermal shock resistance, energy damage theory, splitting test, machine learning

## Abstract

The thermal shock resistance (TSR) of refractories is a critical determinant of the service life and operational safety of high-temperature industrial equipment in metallurgy, building materials, and chemical engineering. This paper systematically reviews the state-of-the-art research on the evaluation of TSR for refractories. On the theoretical level, the evolutionary logic from classical thermoelastic theory to energy-based damage theory, brittleness evaluation criteria, and the dimensional analysis-based R_Π_ theory is delineated, with a comparative analysis of the applicability of various criteria in dense versus porous material systems. Regarding evaluation methodologies, the strengths and limitations of conventional thermal cycling tests, splitting tests (notably Brazilian and wedge splitting), and specialized techniques such as ultrasonic pulsing and nano-indentation are scrutinized. Furthermore, the application of non-destructive monitoring technologies, such as Digital Image Correlation (DIC) and Acoustic Emission (AE), for in-situ damage capture is discussed. Additionally, the potential of machine learning in performance prediction and inverse material design is explored. Finally, it is posited that future research should focus on promoting the development of multiscale, standardized, and intelligent evaluation frameworks to meet the requirements of harsh operating environments in emerging fields such as green metallurgy.

## 1. Introduction

Refractories are critically important to the performance and safety of thermal equipment across high-temperature industrial sectors, ranging from pyrometallurgy and non-ferrous smelting to chemical engineering [1,2]. Refractories are made of aggregates and a matrix similar to those of concrete structures, such materials mainly containing Al_2_O_3_, MgO, CaO, SiO_2_, SiC, Si_3_N_4_, carbon, and so on to withstand high-temperature environments. The thermomechanical reliability of refractories directly affects the service life of industrial furnaces, thermal efficiency, and systemic safety of high-temperature reactors. With the development of green and clean steelmaking technology, refractory linings are increasingly subjected to rigorous service conditions such as severe thermal cycling and abrupt temperature gradients, bringing about substantial internal thermal stresses [3,4]. Once the localized stress concentration exceeds the fracture toughness or fracture energy threshold of refractories, it triggers a cascade of micro-crack initiation, coalescence, and multiscale unstable propagation. This degradation path contributes to the final macroscopic spalling or catastrophic structural failure, deteriorating both production continuity and the stringent requirements for melt cleanliness [5]. Industry production practices show that thermal-shock-induced damage in critical components including ladle linings, nozzles, and slide gates remains the predominant bottleneck limiting the service longevity and operational safety of high-temperature metallurgical equipment [6,7].

**Figure 1:**
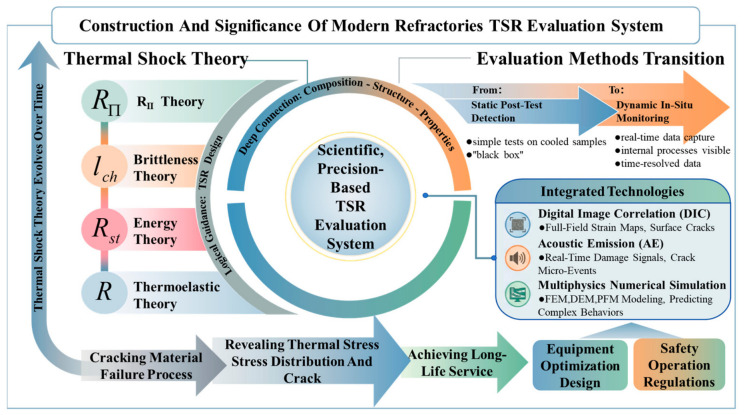
Development of the modern evaluation system for refractories.

Establishing a scientific and precise evaluation system for thermal shock resistance (TSR) is the precondition for evaluating material damage processes and achieving extended service life. The profound significance of the evaluation lies not only in the deep understanding of the physical responses of refractories under extreme temperature gradients, but also in revealing the essential mechanisms of thermal stress distribution and dynamic crack evolution through the deep correlation of composition–structure–performance [8]. To achieve this goal, researchers have conducted extensive and productive theoretical studies and application investigations over recent decades, leading to substantial progress in the characterization and evaluation of TSR [9]. The development evolved from Kingery’s classical thermoelastic theory [10] in the 1950s, based on elasticity, which proposed R-series fracture factors centered on critical stress criteria. It then progressed to Hasselman’s introduction of fracture mechanics and energy balance criteria, constructing an energy damage theory capable of describing the competition between crack initiation and propagation in quasi-brittle materials, along with key criteria such as $R^{\prime \prime \prime \prime }$ and $R_{\mathrm{st}}$ [11]. Subsequently, researchers further developed indirect tensile testing methods, such as the Wedge Splitting Test (WST) and the Brazilian test [12], introducing parameters like specific fracture energy ($G_{f}$) and characteristic crack length ($l_{ch}$) to modify the characterization of material brittleness [13]. By combining with in-situ monitoring technologies such as Digital Image Correlation (DIC) and Acoustic Emission (AE) [14], a technological leap has been achieved, advancing from traditional black-box residual property testing to the in-situ, dynamic capture of the entire damage evolution process. DIC and AE are utilized to transition from “black-box” post-test measurements to dynamic, in-situ monitoring. DIC provides full-field strain maps to capture surface crack evolution, while AE captures elastic stress waves to monitor internal micro-events. However, DIC is limited to surface observations and AE is highly sensitive to environmental noise. Entering the 21st century, the R_Π_ theory based on dimensional analysis provided a novel criterion for TSR evaluation [15]. Furthermore, Machine Learning (ML) now enables the auxiliary prediction of complex damage behaviors [16], making the transition from static post-test detection to dynamic in-situ monitoring possible. Together, these advancements have constructed a contemporary multiscale evaluation system for refractories, ranging from micro defect design to macro life assessment, as demonstrated in Figure 1, providing indispensable underlying data support for the optimized design and safety operation protocols of high-temperature equipment.

Based on the above, this paper systematically reviews the research development and latest advancements in the evaluation of TSR for refractories. First, starting from the logic of theoretical evolution, the application boundaries and limitations of various criteria—ranging from classical thermoelastic criteria to energy damage theory, brittleness assessment, and R_Π_ theory—are comparatively demonstrated and analyzed. Subsequently, the merits and drawbacks of advanced characterization techniques, such as conventional quenching methods, splitting tests, and non-destructive testing (NDT), are reviewed in detail, followed by an in-depth exploration of the cutting-edge application of ML in property prediction. Finally, in light of industrial digitalization trends, this paper proposes outlooks on the standardization of evaluation systems, multiscale correlation, and intelligent damage early-warning. This work aims to provide a theoretical reference for the precision design and engineering application of next-generation high-performance refractory materials, such as those maintaining a residual strength retention rate of >80% under severe thermal cycling (ΔT > 1000 °C), demonstrating stable crack arrest capabilities, and exhibiting extended fatigue life in multi-field coupled extreme environments.

## 2. Research Progress in Thermal Shock Resistance Theory

### 2.1. Thermoelastic Theory (Critical Stress Fracture Theory)

**Figure 2:**
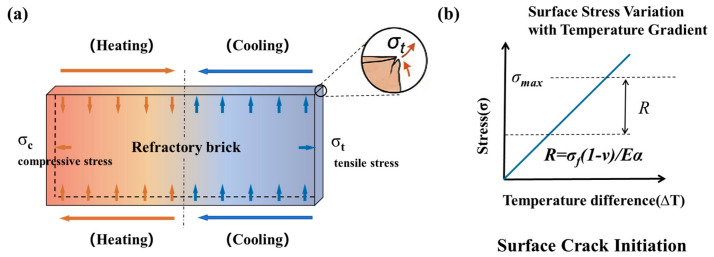
Schematic of thermoelastic theory: (**a**) schematic of crack initiation due to thermal shock; (**b**) variation of stress with temperature gradient.

In the 1950s, Kingery [10,17] proposed the critical stress fracture theory based on the principles of elasticity. This theory employs the thermal stress ($\sigma_{H}$) induced by a temperature difference (ΔT) and the inherent strength of the material ($\sigma_{f}$) as the core criteria for evaluating TSR. According to this theory, if the thermal stress ($\sigma_{H}$) generated by the thermal shock does not exceed the ultimate strength, the refractories remain undamaged. However, once the thermal stress ($\sigma_{H}$) resulting from the maximum temperature difference reaches or surpasses the inherent strength (i.e., fracture strength), crack initiation occurs, ultimately leading to structural failure. The schematic illustration of refractory cracking in the presence of thermal stress is shown in Figure 2a. To quantitatively evaluate the TSR, the first, second, and third thermal stress resistance factors (R, $R^{\prime }$, and $R^{\prime \prime }$) have been established, as expressed in Equations (1), (2), and (3), respectively.

(1) $$R=\Delta {T\frac{\sigma f(1-\nu )}{E\alpha }}_{max}$$

(2) $$R^{\prime }=\lambda R=\lambda \times \frac{\sigma f(1-\nu )}{E\alpha }$$

(3) $$R^{\prime \prime }=\frac{1}{\rho c_{p}}\cdot R^{\prime }=\frac{\lambda }{\rho c_{p}}\cdot R=aR$$

In these equations, $\sigma_{f}$ represents the fracture strength (intrinsic strength) of the material, ν is Poisson’s ratio, α is the coefficient of thermal expansion (CTE), E is Young’s modulus, λ is the thermal conductivity, ρ is the density, $c_{p}$ is the specific heat capacity, and a is the thermal diffusivity. The term $\Delta T_{max}$ denotes the critical temperature difference at which the thermal stress equals the fracture strength, serving as the first thermal stress resistance factor (R). The first factor (Equation (1)) indicates that a larger temperature gradient induces higher internal stress (Figure 2b). This factor is suitable for evaluating the TSR of refractories under conditions of instantaneous quenching or heating, where heat dissipation effects are neglected. To explain these effects, thermal conductivity (λ) was incorporated into the parameters to derive the second thermal stress resistance factor ($R^{\prime }$, Equation (2)), which assesses TSR under conditions of slow cooling or heating. Furthermore, since different materials exhibit vastly different temperature gradients and thermal stresses even at identical cooling/heating rates, thermal diffusivity (a), which represents the material’s ability to achieve thermal equilibrium, was introduced into the second factor to yield the third thermal stress resistance factor ($R^{\prime \prime }$, Equation (3)). The core criteria and applicability of each factor within Kingery’s thermoelastic theory are compared in Table 1. The equations indicate that superior TSR requires a combination of high thermal conductivity and tensile strength, alongside a low CTE and Young’s modulus. These formulas assume a flawless initial state (free of pores and cracks). However, the microstructure of real refractories contains inherent defects such as microcracks and micropores. Consequently, the R, $R^{\prime }$, and $R^{\prime \prime }$ factors often fall short in accurately evaluating the TSR of complex refractory systems.

Based on the three aforementioned thermal stress fracture factors, researchers have conducted extensive TSR evaluations in recent years. For instance, Wang et al. [18,19] fabricated BNNTs/Si_3_N_4_ composites with varying weight fractions (0~2 wt%) using the vacuum hot-pressing sintering method. Utilizing Kingery’s thermal shock fracture theory, they constructed models for the first and second thermal shock factors (*R* and *R*′) for boron nitride nanotube (BNNT) strengthened and toughened ceramic composites to evaluate their TSR. Experimental results under thermal cycling (rapid cooling and heating) conditions indicated that as porosity increased, the material strength significantly decreased, leading to a progressive reduction in the first thermal shock factor (*R*). When porosity remained below 10%, the *R* values were relatively stable, suggesting that a moderate amount of porosity within the ceramic can effectively absorb the energy of crack propagation, thereby maintaining a certain level of thermal shock fracture resistance. The authors further evaluated the TSR of the BNNTs/Si_3_N_4_ composites under slow heating and cooling conditions using the second thermal shock factor model (*R*′), elucidating the mechanism of improved TSR through the cumulative cyclic crack propagation mechanism. The results demonstrated an approximately linear relationship between the *R*′ factor and the thermal shock temperature difference (ΔT) of refractories. As the temperature increased, the *R*′ factor declined linearly, specifically, a higher thermal shock temperature corresponded to a smaller *R*′ value and lower TSR. The cumulative crack propagation length (Δ*L*) was found to be inversely proportional to the *R*′ factor, indicating that the longer the Δ*L*, the smaller the *R*′ value. Consequently, at a fixed operating temperature, enhancing the fracture toughness and minimizing the cumulative cyclic crack propagation length are essential prerequisites for improving the TSR.

### 2.2. Energy Theory (Thermal Shock Damage Theory)

**Figure 3:**
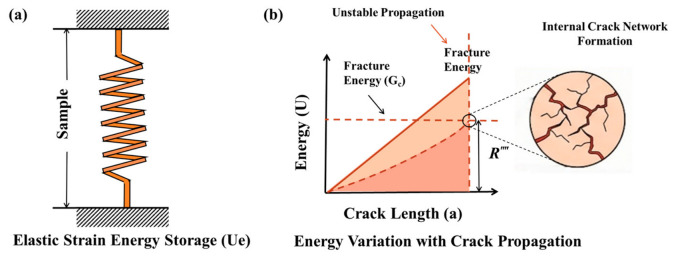
Schematic of thermal shock damage theory: (**a**) schematic of elastic strain energy storage; (**b**) variation of energy with crack propagation.

From the perspectives of energy theory and fracture mechanics, Hasselman [11,20,21] proposed the thermal shock damage theory, which assumes the existence of initial microcracks within the material. This theory establishes the balance between thermoelastic strain energy (*W*) and fracture energy (*G*) as the criterion for thermal shock failure, as illustrated in Figure 3a. In this framework, the thermoelastic strain energy is stored like a spring, and thermal shock damage is attributed to the initiation and propagation of cracks. Under various temperature conditions, refractories undergo a dynamic process of crack nucleation, propagation, and arrest. Not all crack nuclei lead to ultimate fracture; the driving force for crack propagation is provided by the elastic energy stored at the fracture site, while the effective surface energy required to create new crack surfaces constitutes the energy dissipation mechanism that inhibits propagation. Figure 3b shows the energy variations as a function of crack propagation. When the release of elastic strain energy (*W*) induced by thermal stress exceeds the energy required for crack nucleation and the creation of new surfaces (*G*), cracks will nucleate and propagate continuously, eventually leading to catastrophic failure of refractories. Depending on the initial crack length and the severity of the thermal shock, Hasselman proposed four crack propagation resistance factors ($R^{\prime \prime \prime }$, $R^{\prime \prime \prime \prime }$, $R_{st}$, and $R_{st}^{\prime }$).

(4) $$R^{\prime \prime \prime }=\frac{E}{\sigma_{f}^{2}(1-\nu )}$$

(5) $$R^{\prime \prime \prime \prime }=\frac{GE}{\sigma_{f}^{2}(1-\nu )}$$

(6) $$R_{st}=\sqrt{\frac{G}{\alpha^{2}E}}$$

(7) $$R_{st}^{\prime }=\lambda \cdot \sqrt{\frac{G}{\alpha^{2}E}}$$

where v represents Poisson’s ratio, α is the coefficient of thermal expansion, E denotes Young’s modulus, λ is the thermal conductivity, $\sigma_{f}$ is the inherent strength of the material, and G refers to the fracture surface energy.

Both $R^{\prime \prime \prime }$ and $R^{\prime \prime \prime \prime }$ serve as thermal shock damage resistance parameters for materials characterized by initial short cracks. Specifically, $R^{\prime \prime \prime }$ focuses on elastic strain energy, rendering it suitable for comparing materials with similar fracture surface energies. In contrast, $R^{\prime \prime \prime \prime }$ accounts for both elastic strain and fracture surface energy, facilitating a more comprehensive evaluation of materials with divergent fracture properties. Based on energy-balance and fracture mechanics theories, decreasing the intrinsic strength ($\sigma_{f}$) while increasing the fracture surface energy (G) effectively enhances a material’s resistance to crack propagation.

While $R^{\prime \prime \prime }$ and $R^{\prime \prime \prime \prime }$ focus on short cracks, the parameters $R_{st}$ and $R_{st}^{\prime }$ are tailored for scenarios involving initial long cracks, with $R_{st}^{\prime }$ representing more severe thermal shock conditions. A comparative analysis between critical stress fracture theory (Equations (1)–(3)) and thermal shock damage theory (Equations (4)–(7)) reveals seemingly contradictory conclusions. This discrepancy arises from their distinct theoretical foundations: the former is primarily applicable to dense, high-strength ceramics, whereas the latter is better suited for porous, low-strength refractories.

The process of thermal shock damage typically evolves through four stages: crack nucleation, micro-crack initiation, propagation, and ultimate failure. While crack nucleation governs the incipient damage stage, propagation becomes the decisive factor in the final failure phase. However, Hasselman’s theory still requires refinement, particularly concerning the stochastic nature of initial crack lengths and the temperature-dependent variability of thermophysical properties. Table 2 summarizes the core criteria and applicability of these various factors within the framework of Hasselman’s theory.

### 2.3. Brittleness Evaluation Theory

Harmuth [13] assumed that most refractories endure simultaneous mechanical and thermal stresses during service. Under these complex stress conditions, numerous cracks initiate within the material, yet their propagation remains relatively limited. Based on Hasselman’s thermal shock damage theory, the concept of characteristic crack length ($l_{ch}$) was introduced. Furthermore, he derived a dimensionless brittleness parameter (B) by incorporating material dimensions. The required parameters for Equations (8) and (9) are determined via the wedge splitting test (WST) as follows. Consequently, both $l_{ch}$ and B serve to quantify the resistance to crack propagation of refractories.

(8) $$l_{ch}=\frac{G_{f}\cdot E}{f_{t}^{2}}$$

(9) $$B=\frac{f_{t}^{2}\cdot L}{G_{f}\cdot E}=\frac{L}{l_{ch}}$$

where $G_{f}$ denotes the specific fracture energy, $f_{t}$ the tensile strength, L the specimen length along the crack propagation direction, and E the elastic modulus.

In brittleness evaluation theory, a higher $l_{ch}$ value generally indicates a larger fracture process zone (FPZ). This requires greater energy dissipation before propagation, ensuring more stable crack growth. Consequently, high $l_{ch}$ values directly correlate with superior crack propagation resistance and enhanced TSR. Conversely, the brittleness index (B) is a dimensionless parameter quantifying the degree of brittleness. Lower B values signify relatively higher toughness and improved TSR. The core of this theory lies in quantifying brittle behavior through parameters such as $G_{f}$ and $l_{ch}$. Based on the energy balance criterion, the theory assumes that crack initiation and propagation under thermal shock depend on the interplay between elastic strain energy and fracture surface energy. Parameters $G_{f}$ and $l_{ch}$, obtained via wedge splitting tests, allow for the direct calculation of Hasselman’s damage factors (e.g., $R^{\prime \prime \prime }$ and $R_{st}$). This enables a modified evaluation of crack propagation resistance in porous and low-strength refractories. However, the model requires further refinement due to inconsistent initial crack lengths and temperature-dependent material properties.

### 2.4. RΠ Theory Based on the Π Theorem

The Buckingham Π theorem, proposed by E. Buckingham in 1914, constitutes the foundational theory of dimensional analysis. This theorem states that a physical problem involving n physical variables and k independent fundamental dimensions can be simplified. Specifically, the relationship among these variables is reducible to a functional form comprising n-k independent dimensionless parameters (or Π terms). Equation (10) represents this simplified relationship [22].

(10) $$F(\Pi_{1},\Pi_{2},\ldots ,\Pi_{p})=0$$

The Buckingham Π theorem identifies intrinsic relationships between variables without solving specific differential equations. It extracts the core dimensionless parameters governing physical phenomena. This theorem significantly simplifies experimental data processing and provides a guiding framework for experimental design and theoretical modeling. Currently, theoretical reports using the Buckingham Π theorem to characterize TSR are still insufficient. Nevertheless, scholars have applied this theorem to investigate the TSR of alumino-silicate refractories. Qin et al. [15] from Wuhan University of Science and Technology developed a combination of dimensionless parameters, as presented in Equation (11), for comprehensive stability evaluation. Their research converts multiple physical variables into three independent dimensionless groups. A weighted sum of these groups yields a comprehensive evaluation index (R_Π_) to determine the TSR of refractories.

(11) $$R_{\Pi }=u\Pi_{1}+v\Pi_{2}+w\Pi_{3}$$

where Π_1_, Π_2_, and Π_3_ denote the dimensionless parameters. The variables u, v, and w represent the real scalar coefficients, while R_Π_ serves as the comprehensive evaluation index.

Utilizing the Π theorem, the study integrates multiple TSR variables into three distinct dimensionless terms. Equations (11), (12), and (13) provide the specific formulations for Π_1_, Π_2_, and Π_3_, respectively.

(12) $$\Pi_{1}=\frac{\sigma_{f}}{E}$$

(13) $$\Pi_{2}=\frac{\gamma_{\mathrm{wof}}A_{Q}}{EV}$$

(14) $$\Pi_{3}=\frac{\lambda \rho^{0.5}}{E^{0.5}\alpha \gamma_{wof}}$$

where α denotes the coefficient of thermal expansion (CTE), λ the thermal conductivity, E the elastic modulus, and $\sigma_{f}$ the flexural strength. Furthermore, $\gamma_{\mathrm{wof}}$ represents the work of fracture, $V/A_{Q}$ the volume-to-surface area ratio, and ρ the bulk density.

A weighted sum of the three Π terms yields the following comprehensive evaluation index:

(15) $$R_{\Pi }=\frac{u}{i}\frac{\sigma_{f}}{E}+\frac{v}{j}\frac{\gamma_{\mathrm{wof}}A_{Q}}{EV}+\frac{w}{k}\frac{\lambda \rho^{0.5}}{E^{0.5}\alpha \gamma_{wof}}$$

where i, j, and k represent non-zero real numbers.

Based on this theory, Qin et al. [15] conducted physical property tests on four types of alumino-silicate bricks (AL38, AL45, LZ55, and LZ65). Each type was divided into two groups of different sizes (Group S and Group L). The corresponding R_Π_ values were calculated based on the dimensionless parameter expressions, as shown in Table 3. To verify the reliability of R_Π_, the residual strength ratio (the ratio of flexural strength before and after thermal shock) was measured. Under cyclic thermal shock conditions, experimental results demonstrate that R_Π_ values exhibit a clear size effect. For the same brick type, small-sized specimens show superior TSR compared to large-sized ones. For samples with alumina content between 45% and 65%, a higher residual strength ratio correlates with a larger R_Π_ value. This consistency indicates that the R_Π_ theorem can effectively characterize the TSR of refractories under specific conditions. However, for the AL38 sample (38% alumina), the R_Π_ value is relatively high despite a low residual strength ratio. This discrepancy stems from the high content of glassy phase and quartz, which results in a low work of fracture. Consequently, the R_Π_ theorem requires cautious application when evaluating refractories with high glassy phase content. Within a specific composition range, however, the R_Π_ theorem serves as a reliable tool for characterization. Dimensional analysis thus enables a rapid assessment of thermal shock performance in refractories.

The introduction of R_Π_ theory overcomes the limitations of traditional criteria in evaluating multiphase complex systems. Its core advantage lies in the dimensionless terms (Π_1_, Π_2_, Π_3_) extracted via the Buckingham Π theorem. These terms effectively shield against interference from fluctuations in absolute material parameters, enabling cross-system comparisons (e.g., between acidic and basic refractories). Compared with traditional single-factor criteria, R_Π_ theory leverages its dimensionless nature to mitigate absolute value fluctuations. Through the refined adjustment of weighting coefficients (*u*, *v*, *w*), researchers can quantitatively identify the specific contribution of thermal conductivity (λ) and work of fracture (γ_wof_) to system stability under particular operating conditions. Consequently, this provides a theoretical framework for the precision design of refractories based on the “composition–structure–performance” paradigm.

## 3. Evaluation Method of Thermal Shock Resistance

### 3.1. Thermal Cycling Test

**Figure 4:**
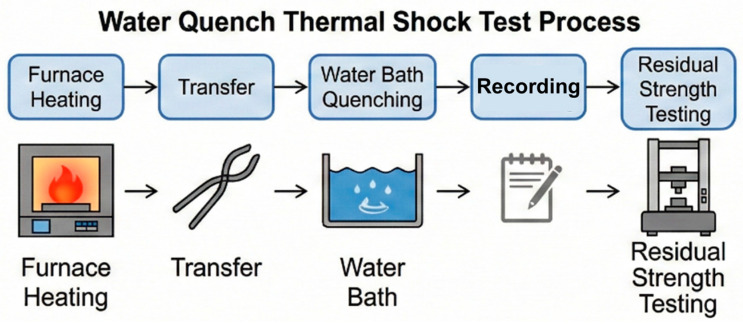
Flowchart of Traditional Water Quenching Method.

The thermal shock cycle test is the most common method for evaluating the TSR of carbon-containing refractories. Its core principle involves simulating the rapid heating and cooling processes experienced during actual service. Figure 4 illustrates the traditional water quenching method. Typically, specimens are heated to a predetermined temperature (usually above 900 °C) and held until thermal equilibrium is reached. Subsequently, the specimens are rapidly removed and immersed in a cooling medium to reach room temperature, completing one thermal shock cycle. The choice of quenching medium, such as air, water, or oil, depends on the specific material characteristics. Water quenching offers a higher cooling rate, while air cooling is slower. Generally, refractories prone to hydration require air or oil as the quenching medium. For quantitative evaluation, operations must strictly follow standards such as GB/T 30873-2014 to ensure the comparability of residual strength retention data across different laboratories. Additionally, attention must be paid to the surface effect during water quenching, specifically the significant influence of the heat transfer coefficient (h) on the distribution of crack initiation.

**Figure 5:**
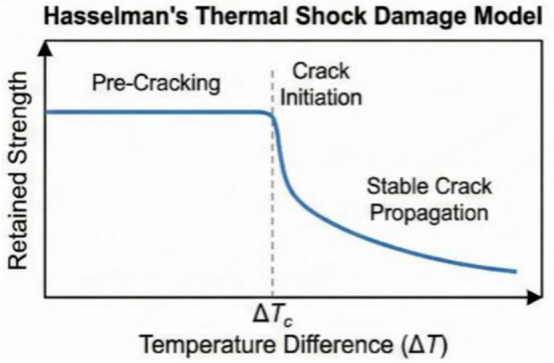
Theoretical curve of residual strength versus temperature difference (Hasselman model).

Quantitative evaluation of TSR typically involves measuring changes in physical properties, such as flexural strength, elastic modulus, or ultrasonic velocity attenuation, before and after thermal shock. These measurements provide a baseline for quantifying damage and stability. Generally, the residual strength retention rate serves as the primary indicator; a higher retention rate signifies superior stability. The theoretical relationship between residual strength and temperature difference is illustrated in Figure 5. As ΔT exceeds the critical temperature difference ($\Delta T_{c}$), cracks initiate. Further increases in ΔT lead to stable crack propagation, resulting in a sharp decline in residual strength and final failure. Alternatively, stability can be characterized by the number of cycles required for the appearance of visible macro-cracks, where more cycles indicate better performance [23]. Wei et al. [24] utilized air quenching to evaluate Y_2_O_3_-doped ceramics; samples were heated to 1100 °C, held for 30 min, and then rapidly cooled with compressed air for 5 min over three cycles. The results showed that Zr(OH)_4_ doping significantly increased the residual strength ratio. At a 3 wt% Zr(OH)_4_ concentration, the retention rate reached 54.5%. This improvement likely stems from increased porosity, which alters energy release paths and inhibits crack propagation. Li et al. [25] employed water quenching for mullite-corundum castables. After treatment at 1500 °C, specimens were held at 1100 °C for 0.5 h and quenched in water for three cycles. The addition of 0.8 wt% nano-ZrO_2_ yielded the highest retention rate (38.1%). This is attributed to the energy dissipation effect of microcracks, which are induced by the tetragonal-to-monoclinic (t phase to m phase) phase transformation of nano-ZrO_2_ alongside the crack-pinning effect of the interlocking structure formed by plate-like CA_6_ and in-situ mullite. Yuan et al. [26] also used air quenching for testing dense periclase–forsterite composite aggregates; the samples calcined at 1600 °C exhibited a retention rate of 115%, indicating a performance enhancement post-thermal shock. This is primarily due to the dense interfacial structure formed by forsterite-coated periclase, which reduces overall thermal conductivity and thermal stress. Furthermore, the liquid phase filling pores at high temperatures enhances the stability. Xu et al. [27] used oil quenching to compare magnesia-carbon (MgO-C) refractories containing flake graphite (GF) and granulated graphite (GP). After treating at 1400 °C, both types showed similar initial strengths. However, after five cycles of oil quenching from 900 °C, the GP specimens achieved an 81.04% retention rate, surpassing the 75.37% of the GF specimens. This indicates that granulated graphite better enhances the TSR of MgO-C refractories. As the most common standardized method, the heating-cooling cycle test is characterized by its procedural simplicity, rigorous conditions, and intuitive results. It is highly effective for most dense refractories, providing a rapid assessment of TSR under extreme thermal cycling conditions.

The primary disadvantage of rapid quenching lies in the sharp increase of internal stress due to excessive cooling rates, which often triggers deformation or cracking. This method imposes constraints on the shape and dimensions of the workpiece, with higher risks for complex or thin-walled components. Furthermore, the selection and control of the quenching medium are critical; improper operation may lead to non-uniform hardness or substandard performance. Additionally, a subsequent tempering (or stress-relief) treatment is typically required to eliminate residual stresses, thereby increasing both process complexity and costs.

### 3.2. Splitting Test

#### 3.2.1. Brazilian Test

The Brazilian splitting method was first proposed in 1943 by Carneiro [28] and Akazawa [29]. Initially developed for geotechnical testing, the method was later standardized by the International Society for Rock Mechanics and the American Society for Testing and Materials [12,28,29]. The procedure involves placing a cylindrical or disc-shaped specimen in a testing machine and applying diametrical compression. This loading generates a uniform tensile stress perpendicular to the loading direction. When this stress reaches the maximum tensile strength, the specimen undergoes splitting failure along the loaded diameter. Under rapid heating and cooling conditions, non-uniform expansion or contraction induces thermal stresses. The resulting damage is primarily caused by tensile failure. Consequently, changes in tensile strength provide the most sensitive reflection of thermal shock damage. The tensile strength measured via the Brazilian test effectively indicates the TSR of refractories, a more significant decline in tensile strength indicates poorer stability.

During the Brazilian splitting test, the regions near the loading points primarily endure compressive stress, while the specimen center remains in a state of tensile stress. To ensure accurate tensile strength measurements, cracks must initiate from the center of the specimen. The theoretical foundation of this method relies on the linear elastic assumption, treating the disc specimen as a homogeneous, isotropic elastic body. The strength calculation formula (Equation (16)) is derived from the analytical stress solution for a disc under linear loading within the framework of elasticity mechanics. In practice, arc-shaped loading jaws (or platens) are typically used to apply the load instead of idealized theoretical line loads. This modification mitigates stress concentration near the loading points. By achieving tensile failure through simple compressive loading, this indirect method overcomes common challenges in direct tensile testing, such as specimen gripping difficulties and parasitic stress concentrations. Consequently, it has become a widely adopted technique for tensile strength testing in engineering. Furthermore, research frequently incorporates digital image correlation (DIC) and acoustic emission (AE) techniques to monitor strain fields and crack evolution in real-time.

(16) $$\sigma_{t}=\frac{2P}{\pi Dt}$$

where $\sigma_{t}$ denotes the tensile strength, P the peak load at failure, D the diameter of the disc specimen, and t the thickness of the disc specimen.

Kaczmarek et al. [30] applied the Brazilian test in conjunction with an enhanced 2P-DIC method to achieve precise measurements of full-field strain and crack length in ceramics. By monitoring the fracture behavior of refractories, they demonstrated that the material exhibits non-brittle fracture at 1200 °C. At this temperature, crack propagation is more gradual with increased branching, contrasting with the sudden brittle failure observed at room temperature. This reveals the positive impact of high temperatures on crack resistance. Darban et al. [31] systematically evaluated the tensile strength of alumina-spinel refractories at 1200 °C using the Brazilian test integrated with DIC technology. By comparing pristine, lab-slag-penetrated, and post-service refractories, they found that post-service refractories reached a maximum tensile strength of 10.7 MPa. This represents an 80% increase over the pristine material and a 52–70% increase over lab-penetrated samples. The findings indicate that corrosion products, such as calcium aluminates, densify the microstructure. This significantly enhances material stiffness and brittleness, revealing changes in thermal shock behavior under actual operating conditions. Wang et al. [32] utilized the Brazilian test combined with image processing for the automated identification and classification of cracks in Al_2_O_3_-MgO ramming mixes and corundum castables post-thermal shock. This approach enables the quantitative analysis of fracture behavior by effectively distinguishing crack types and measuring their geometric features. The study observed that thermal treatment significantly increased crack dimensions and altered propagation paths, indicating increased brittleness. This methodology provides a reliable quantitative tool for evaluating the TSR of refractories.

Brazilian splitting assumes material homogeneity; however, refractories are predominantly multiphase composite systems. During loading, stress concentrates along weak zones such as aggregate-matrix interfaces and pores. This causes the fracture path to deviate from the center diameter, preventing an accurate reflection of the true tensile strength and leading to significant relative errors. As an indirect tensile test based on the linear elastic assumption, the Brazilian test induces a uniform tensile stress field at the specimen center via compressive loading. This model aligns with Kingery’s thermoelastic theory, as both are rooted in classical elasticity. Consequently, the measured tensile strength serves as a key parameter for evaluating crack initiation resistance. The method has evolved into a comprehensive platform integrating DIC and AE. This integration enables the in-situ synchronization of full-field strain evolution and micro-damage signals, facilitating a dynamic correlation analysis of the stress field–crack propagation–energy release process.

#### 3.2.2. Wedge Splitting Test (WST)

Wedge splitting is a mechanical failure process that utilizes a wedge-shaped tool to apply splitting forces to brittle materials. Its principle involves converting vertical downward pressure into horizontal tensile force via a wedge structure. When the local tensile stress exceeds the material’s tensile strength, regular cracks form or fracture occurs along a predetermined direction. Based on fracture mechanics theory, Professor Harmuth [13] introduced the concepts of characteristic length and thermal shock damage factors. He was the first to adapt the WST, originally developed by Professor Tschegg [33] for studying the fracture behavior of rock and concrete, to investigate the fracture behavior of refractories. This method is employed to evaluate the ability of refractories to resist crack initiation and propagation during thermal shock.

The wedge splitting test apparatus primarily consists of a wedge-shaped indenter, two cylindrical rollers, two load-transmission plates, and a base support. During the test, a vertical load is applied at a constant rate and converted into a larger horizontal tensile load via the transmission assembly. The relationship between these loads is defined by Equation (17). The specimen eventually undergoes failure due to tensile stress, yielding a load-displacement curve, from which parameters such as fracture energy and horizontal load are derived. The WST offers distinct advantages in both specimen preparation and testing. Refractories can be cast directly in molds with pre-cast notches, which facilitates the stable propagation of cracks. Mechanically, the transmission device not only converts the force direction but also amplifies the load, resulting in a horizontal force greater than the applied vertical force. This amplification allows for accurate measurements even on testing machines with lower load capacities, effectively reducing equipment requirements. Regarding safety, the test automatically terminates when the load drops to 15% of its peak value. The critical parameters obtained in the test including fracture energy $G_{f}$ and characteristic length $l_{ch}$ serve as quantitative standards for evaluating the TSR of refractories based on energy and brittleness theories.

(17) $$F_{H}=\frac{F_{V}}{2\tan(\alpha /2)}$$

where $F_{H}$ represents the horizontal splitting load, and $F_{V}$ denotes the applied vertical load. The variable α refers to the wedge angle, typically ranging from 10° to 45°. Specifically, a sharper angle of 10–20° is utilized for highly brittle materials, whereas 30–45° is preferred for materials with low brittleness.

Equation (18) provides the formulation for calculating the tensile strength $\sigma_{NT}$ of the material.

(18) $$\sigma_{NT}=\frac{F_{H,max}}{b\cdot h\frac{6y}{h}}$$

where $F_{H,max}$ represents the maximum horizontal load, while b and h denote the projected width and projected height of the fracture surface, respectively. The parameter y refers to the linear distance from the centroid of the fracture surface to the line of action of the horizontal load. The specific fracture energy ($G_{f}^{\prime }$) of refractories is determined by integrating the area under the load-displacement curve obtained during testing, as expressed in Equation (19).

(19) $$G_{f}^{\prime }=\frac{1}{A}\int_{0}^{\delta_{ult}}F_{H}d\delta$$

where $\delta_{ult}$ represents the critical displacement at the moment of fracture, and A denotes the projected area of the fracture surface. As shown in Equation (20), the characteristic length ($l_{ch}$) can be derived from parameters obtained through the wedge splitting test, including the specific fracture energy ($G_{f}^{\prime }$), the nominal tensile strength ($\sigma_{NT}$), and the elastic modulus (E).

(20) $$l_{ch}=\frac{G_{f}^{\prime }\cdot E}{\sigma_{NT}^{2}}$$

Based on the WST, researchers have quantitatively evaluated the TSR and fracture behavior of refractories. Zhu et al. [34] utilized WST to quantify these properties in low-carbon MgO-C refractories. Their results showed that adding nano-carbon sources, such as carbon nanotubes, significantly enhanced performance; the characteristic length reached 167 mm and the specific fracture energy hit 468 J/m^2^, far exceeding the 110 mm of traditional graphite samples. This effectively improved material toughness and TSR. Fruhstorfer et al. [35] employed micro-wedge splitting tests combined with Digital Image Correlation (DIC) to analyze the fracture behavior of quasi-brittle refractories. The study revealed that size effects in micro-testing restricted the development of the FPZ. Consequently, the specific fracture energy decreased significantly from 112.4 N/m to 55.4 N/m, and the brittleness number dropped from 0.414 to 0.194 compared to standard tests. Vargas et al. [36] also integrated WST with DIC to quantify the fracture performance of castable refractories, determining a tensile strength of approximately 1.7 MPa and fracture energies ranging from 62 J/m^2^ to 115 J/m^2^. Pan et al. [37] analyzed the influence of cement content on the fracture behavior of corundum castables. They found that specimens with 10% cement exhibited the highest fracture energy (~600 N/m) and peak load, attributed to the reinforcement of interfaces by an appropriate amount of interlocking calcium hexaluminate (CA_6_). However, cracks primarily propagated through aggregates, indicating significant brittleness. The WST is more than a mechanical test; it serves as a bridge connecting microstructure to Hasselman’s energy theory. The measured specific fracture energy $G_{f}$ and characteristic length $l_{ch}$ (Equations (8) and (9)) are core input parameters for calculating Hasselman’s R″″ and R_st_ factors. By combining WST with DIC, the evolution of the FPZ can be dynamically quantified. This validates the inhibitory effect of micro-crack initiation on the release of elastic strain energy, achieving a transition from static parameter measurement to dynamic damage mechanism revelation.

In Table 4, we summarize and compare the TSR factor values, thermal cycling results and wedge splitting test results of several typical refractories including Al_2_O_3_-C, MgO-C and Al_2_O_3_-MgO refractories. It should be noted that these values normally depend on the microstructure and phase compositions of refractories as well as the testing conditions.

### 3.3. Non-Destructive Testing

#### 3.3.1. Ultrasonic Pulse Velocity

Ultrasonic Pulse Velocity (UPV) is a non-destructive testing (NDT) technique used to evaluate material quality without compromising its structural integrity. While commonly employed for concrete performance testing in the construction industry, where traditional destructive methods are often inefficient and sample-intensive, UPV has developed rapidly in recent years by leveraging the propagation characteristics of acoustic waves to infer internal material states. UPV overcomes the limitations of destructive testing, providing a fast and cost-effective means of quality control. As a simple, sensitive, and reliable NDT technique, it effectively monitors the damage evolution of refractories under thermal shock conditions. The TSR can be evaluated by measuring changes in ultrasonic velocity and calculating the dynamic Young’s modulus. Generally, a greater decrease in ultrasonic velocity indicates more severe internal cracking and poorer TSR. Similarly, a steeper decline in the dynamic Young’s modulus signifies faster stiffness degradation and more acute thermal shock damage [38].

**Figure 6:**
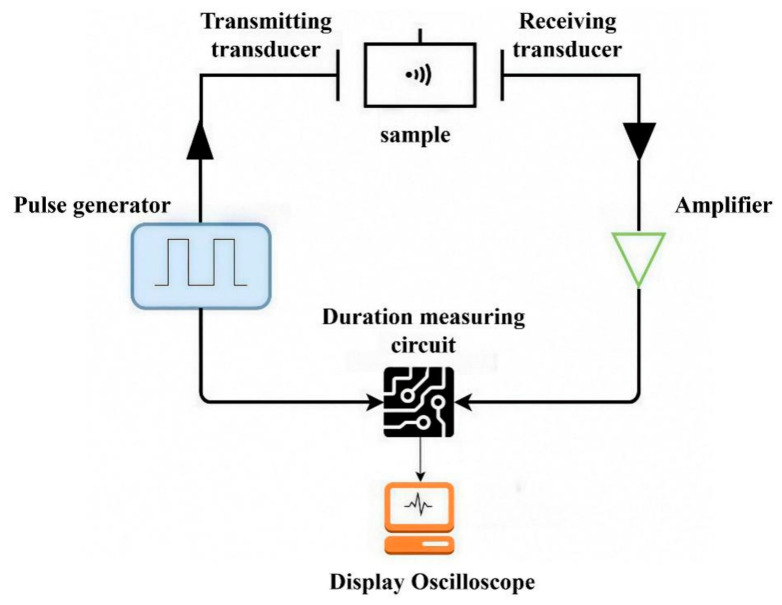
Schematic diagram of the ultrasonic pulse velocity measurement method [39].

Figure 6 illustrates the principle of Ultrasonic Pulse Velocity (UPV) testing, which leverages the propagation characteristics of ultrasonic waves for a comprehensive material assessment. The process begins with a pulse generator producing short, periodic electrical pulses. These are converted by a transmitting transducer into mechanical ultrasonic pulses and introduced into the specimen surface. As waves propagate through the interior, their velocity is closely correlated with the density and compressive strength of the material: higher density and strength result in faster wave propagation. Conversely, internal defects such as cracks or voids cause velocity reduction or energy attenuation. Furthermore, ultrasonic waves undergo refraction and reflection at phase interfaces and defect sites, generating various waveforms including longitudinal and transverse waves. Longitudinal waves, being the fastest, arrive first at the receiving transducer, which reconverts the ultrasonic signal into an electrical signal. After amplification, an integrated electronic timer precisely measures the travel time, displayed in real-time on an oscilloscope. By analyzing the time interval between transmission and reception relative to the known specimen length, the pulse velocity can be calculated. High-quality, dense materials exhibit higher velocities, while those with discontinuities like pores and cracks show a significant drop in velocity [39]. Marie [40] utilized UPV to track performance trends in concrete with rubber contents ranging from 0% to 100%. Based on the results, the performance degradation was categorized into three zones: A, B, and C. In Zone A (0–25%), the UPV values remained high, indicating good quality; although the compressive strength decreased, it remained within an acceptable range. Consequently, a 25% rubber content was identified as the optimal dosage, balancing resource utilization with mechanical integrity. Furthermore, real-time UPV monitoring during axial compression revealed that as rubber content increased, the slope of UPV attenuation under stress became gentler. This indicates enhanced ductility and a retarded crack propagation rate. Ghosh et al. [41] investigated the relationship between the compressive strength of geopolymer concrete and UPV using a high-power pulser. Their results demonstrated a linear correlation between UPV and strength, suggesting that UPV can effectively estimate geopolymer concrete strength with a low standard error of 1.6–2.34 MPa.

**Figure 7:**
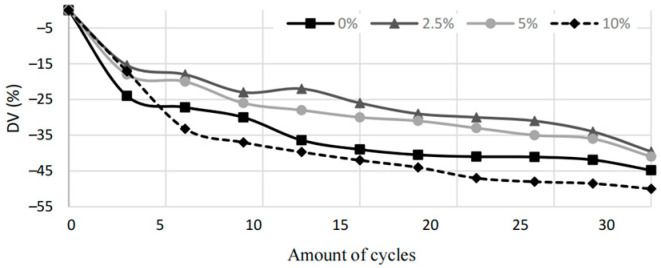
Variation of ultrasonic pulse velocity (DV) of refractory casting materials with different HCM (cylindrical alumina beads) contents during the thermal cycling process [42].

While UPV is widely used in concrete testing, its application in refractories is still emerging. However, some researchers have begun utilizing UPV to assess the performance of refractories after thermal shock cycles. Boccaccini et al. [38] employed UPV for the non-destructive evaluation of damage in two types of cordierite-mullite refractory plates subjected to varying thermal cycles. By measuring the ultrasonic velocity in different directions and calculating the dynamic Young’s modulus, they found that velocities in both the longitudinal and thickness directions decreased with an increasing number of cycles. Correspondingly, the dynamic Young’s modulus also declined. The results indicate that more severe thermal shock damage leads to a greater reduction in both velocity and modulus. This demonstrates a high correlation between UPV technology and thermal shock damage in refractories, allowing for the quantitative characterization of stability and providing effective criteria for industrial guidance. Stonys et al. [42] utilized UPV to measure propagation velocity in refractory castables containing hollow corundum microspheres to detect internal micro-cracks induced by thermal shock. As illustrated in Figure 7, the velocity variation (DV) increased with the number of thermal cycles. Their research suggests that a larger drop in ultrasonic velocity corresponds to a higher density of micro-cracks and other discontinuities formed during cycling, thereby indicating a lower TSR.

The attenuation of ultrasonic velocity in UPV is directly correlated with the degradation of the elastic modulus, providing a viable pathway to map experimental results onto Hasselman’s energy theory. The proven maturity of this method in fields such as concrete, for strength and damage assessment, underscores its significant potential in the refractory industry. Future research should focus on integrating UPV with technologies such as DIC and AE to construct a robust acoustic-parameters–microstructure–macro performance correlation model. This will facilitate the transition of evaluation methodologies toward standardization and intelligent diagnostics.

#### 3.3.2. Digital Image Correlation (DIC)

**Figure 8:**
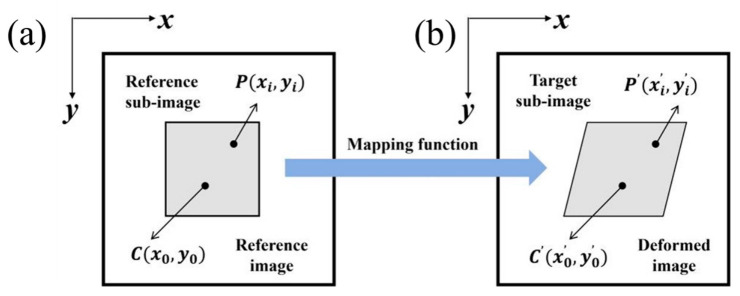
Principle diagram of Digital Image Correlation (DIC) technology: (**a**) Reference image; (**b**) Deformed image [45].

DIC is a non-contact optical measurement technique. By analyzing random speckle patterns on a material’s surface before and after deformation, the method tracks the movement of homologous points to achieve precise full-field quantification of displacement and strain distributions. DIC was independently proposed in the 1980s by the Japanese scholar Yamaguchi [43] and Peters and Ranson [44] at the University of South Carolina, as illustrated in Figure 8a,b. Based on digital image processing, it compares gray-scale intensity distributions between the initial and deformed states. Using algorithms such as subset matching, it calculates the local displacement field, from which the strain field is derived to characterize the crack evolution process [45]. The whole system mainly contains an image acquisition system (resolution: 4096 (H) × 3000 (V) pixels, maximum full-frame rate: 30 fps), blue light high-brightness monochrome light source (eliminate high-temperature thermal radiation interference, central wavelength: 450 nm, output power: 80 W/continuous mode, 120 W/pulse mode, pulse time width: 2.9 ms) and narrowband filter (central wavelength: 450 nm, bandwidth: 20 nm, transmittance: 90%). The baseline noise/error before testing should be eliminated to improve the SNR (Signal-to-Noise Ratio) and avoid reconstruction distortion. In refractory research, DIC is instrumental in visualizing the formation and expansion of the FPZ, tracing crack propagation paths, and investigating strain evolution laws under chemo-mechanical coupling in high-temperature corrosive environments. Under identical thermal shock temperature gradients, an earlier appearance, wider distribution, and higher magnitude of strain concentration observed via DIC indicate weaker resistance to thermal shock and poorer stability. Due to its non-intrusive nature, DIC is exceptionally suited for real-time monitoring in harsh environments involving high temperatures and large strains. Integrating DIC with traditional wedge splitting tests (WST) enables accurate capture of the dynamic crack evolution process.

Wan et al. [46] employed DIC in conjunction with the Brazilian test to systematically analyze the fracture behavior of lightweight mullite-SiC refractories coated with glass-ceramics. By monitoring crack propagation and strain field distributions, they observed that all specimens developed micro-cracks due to horizontal tensile stress prior to reaching the peak load (F_H, max_), which enhanced the fracture performance. Compared to uncoated lightweight aggregate samples (M-50), the coated specimens (M13-50 and M15-50) exhibited more complex propagation paths and an increased number of crack branches. This confirms that the glass-ceramic coating strengthened the interfacial bonding between the porous mullite aggregates and the matrix. Consequently, cracks underwent deflection and branching during propagation, consuming more energy and improving fracture toughness. Furthermore, the coated samples displayed more uniform strain distributions with reduced local stress concentration, effectively delaying the initiation and rapid expansion of the macro-crack. Dai et al. [47] combined DIC with the WST to observe and quantify the FPZ in industrial magnesia and magnesia-alumina spinel refractories. The DIC method successfully captured the development of surface strain fields. The results showed that the magnesia-alumina spinel material contained a micro-crack network, resulting in a larger FPZ and superior energy dissipation capacity. In contrast, the pure magnesia material exhibited no significant FPZ, indicating higher brittleness and a fracture behavior closer to linear elasticity. Notably, the FPZ in the spinel material began forming before the peak load and expanded as loading progressed until macro-crack initiation. However, in pure magnesia, the macro-crack localized immediately at the peak load, with very limited FPZ development. These findings underscore that the presence of micro-crack networks grants refractories superior fracture toughness and crack resistance, providing a robust theoretical basis for evaluating TSR.

Belrhiti et al. [48] combined DIC with the WST to explore the relationship between the mechanical behavior and microstructure of magnesia-alumina spinel and magnesia refractories. Their findings indicate that the TSR of refractories can be significantly improved by optimizing their microstructural characteristics. Fruhstorfer et al. [49] investigated the energy dissipation during WST for two types of alumina refractories with different matrix densities using the DIC-WST integrated approach. The results showed that the material with a denser matrix exhibited a greater delamination width and a higher density of strain concentration points, which led to macro-crack deflection. In contrast, the specimen with a lower matrix density demonstrated a larger FPZ and higher fracture energy, indicating a more robust capacity for energy absorption during the fracture process.

#### 3.3.3. Acoustic Emission (AE)

**Figure 9:**
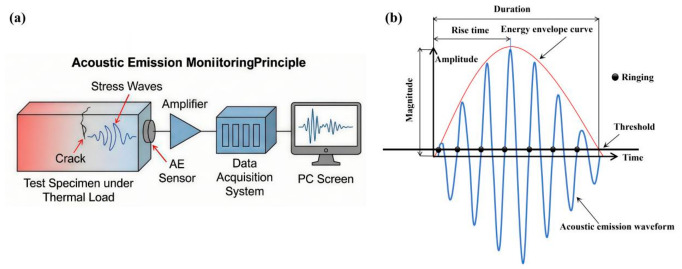
Schematic diagram of the (**a**) monitoring principle of online acoustic emission technology and (**b**) characteristic parameters of acoustic emission [27].

AE is an NDT technique that monitors internal damage development by detecting elastic waves released during micro-damage processes under load. As illustrated in Figure 9a, when cracks initiate and propagate, the technique captures transient waveforms via piezoelectric sensors. Various parameters, including event counts, amplitude distribution, and frequency characteristics, are extracted (Figure 9b) to identify damage types, evaluate damage severity, and predict material failure. Under the same number of thermal shock cycles, a higher frequency of recorded AE signals and greater cumulative energy generally indicate more intense and frequent internal cracking, reflecting poorer TSR. In the field of refractories, AE technology has been widely applied for damage monitoring and failure analysis of brittle materials, such as ceramics and refractories, specifically under conditions of thermal shock, mechanical loading, and thermal cycling. This technique not only enables real-time monitoring of damage accumulation under in-service conditions but also provides a crucial basis for optimizing material design and life prediction. Consequently, it has become a standard tool for evaluating the TSR of refractories [50,51].

Bouchetou et al. [52] synthesized an innovative zirconia-mullite composite by sintering andalusite, alumina, and zircon precursors at 1600 °C. Utilizing non-destructive characterization methods, including ultrasonic pulse-echo and AE, they evaluated its corrosion resistance to alumina-lime slag and soda-lime glass, as well as its TSR. Their findings revealed a unique microstructure with fine zirconia particles uniformly dispersed within a mullite matrix, significantly enhancing both corrosion and TSR. Notably, a self-healing mechanism was observed after the initial thermal shock, where micro-cracks were effectively repaired by the glass phase upon reheating. Yan et al. [53] evaluated the fracture behavior of lightweight magnesia-based refractories using a combination of WST, DIC, and AE. The results indicated that the addition of spinel powder led to a more gradual energy release pattern. Analysis of AE signals showed denser energy clusters in the post-peak region. Furthermore, peak frequency analysis revealed a reduction in the proportion of trans-granular cracks (from 26.5% to 23.3%) and an increase in crack propagation along the aggregate/matrix interface, thereby significantly enhancing crack resistance and reducing brittleness. Xu et al. [54] analyzed the fracture behavior of MgO-C refractories using Brazilian tests integrated with DIC and AE. They monitored the energy release during fracture for various samples: MM (traditional fused magnesia), MG (graphite-coated magnesia), and their respective thermal-shocked versions (MM-TS, MG0.5-TS). Before reaching the peak load, energy was released gradually; however, once the peak load was exceeded, the stored energy was abruptly released, causing a sharp surge in normalized cumulative energy. A higher energy release typically corresponds to more severe local damage. Compared to pristine samples, the thermal-shocked specimens (MM-TS and MG0.5-TS) exhibited reduced energy release during reloading due to increased pre-existing cracks and a loosened matrix, which lowered the fracture energy. Notably, AE signals were only generated when the stress reached the previously applied maximum stress level. The samples with graphite-coated magnesia exhibited a flatter cumulative AE energy curve after thermal shock, indicating more controlled crack expansion and enhanced energy dissipation capacity, which improved the residual strength retention from 56.0% to 62.6%.

### 3.4. Nanoindentation

**Figure 10:**
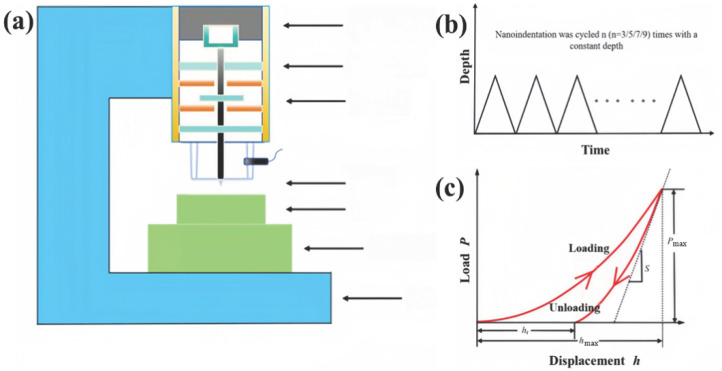
(**a**) Structure of the nanoindentation instrument system, (**b**) Schematic diagram of the cyclic nanoindentation loading process, and (**c**) Nanoindentation load-displacement curve [58].

Nanoindentation is a pivotal experimental method for characterizing the mechanical properties of materials at the nanometer scale. As illustrated in Figure 10a, the apparatus utilizes a specific indenter made of hard materials, such as diamond, to apply precisely controlled micro-loads to the specimen surface while simultaneously monitoring the indentation depth in real-time. Through continuous cyclic loading (Figure 10b), a load-displacement curve is obtained (Figure 10c). Analysis of this curve allows for the calculation of various mechanical parameters, including hardness, elastic modulus, fracture toughness, and creep behavior without the need for direct observation of the residual impression. By analyzing key parameters such as the elastic modulus and fracture toughness, researchers can effectively assess thermal shock damage and evaluate the overall TSR of the material. Due to its high displacement resolution, minimal sample requirements, and ability to test microscopic regions, this technology is widely used for films, coatings, biomaterials, and microelectronic devices [55]. Liang et al. [56] employed nanoindentation to study the variation in mechanical properties of laser solid-formed Ni-based superalloys along the repair height. Their results showed that the elastic modulus followed a trend of increasing and then decreasing with height, while the hardness in the stray grain zone was lower than that of the matrix and decreased along the build direction, revealing the critical role of microstructural gradients in local plastic deformation. Guo et al. [57] utilized nanoindentation to investigate the indentation creep behavior of plasma-sprayed nanostructured LCZ coatings. By analyzing the relationship between indentation depth and time, they found that LCZ coatings exhibit superior creep resistance compared to traditional 8YSZ coatings.

Optimizing the macroscopic mechanical properties of materials fundamentally relies on microstructural design to achieve the reinforcement of microscopic mechanical performance. For refractories, microscopic mechanical properties are critical for resisting micro-crack initiation and propagation. Therefore, acquiring these microscopic parameters is of great significance for deepening research into TSR and establishing the intrinsic link between microstructure and macroscopic performance. Among various methods, nanoindentation has emerged as a key approach for accurately quantifying the microscopic mechanical parameters of materials due to its exceptional micro-scale probing capabilities. However, measuring the micro-nano mechanical parameters of refractories using this technique presents numerous challenges. This is primarily because nanoindentation results are highly sensitive to microstructural variations, while refractories are characterized by complex compositions, coarse structures, and abundant defects, exhibiting significant non-homogeneity. These factors make it difficult to obtain stable and reliable experimental data [59]. Despite these challenges, researchers have achieved significant breakthroughs. Feng et al. [60] utilized nanoindentation to analyze the specific fracture energy of the matrix in low-carbon Al_2_O_3_-C refractories reinforced with CNTs/MgAl_2_O_4_ whiskers, based on load-displacement curves. The results demonstrated that the specific fracture energy of the sample containing 3.0 wt% composite reinforcement increased by 29.4% compared to the control sample. From a micromechanical perspective, this confirms that CNTs/MgAl_2_O_4_ whiskers significantly strengthen the refractory matrix through bridging and crack deflection effects, as well as by promoting micro-cracking. These mechanisms effectively absorb energy during thermal shock and arrest crack propagation, thereby enhancing the TSR of the material.

Nanoindentation achieves the integration of micromechanical response with macroscopic damage theory. Through high-resolution load-displacement curves, this technique directly quantifies the hardness, elastic modulus, and fracture toughness of micro-phases, including the matrix, grain boundaries, and secondary phases. These parameters form the physical foundation for understanding the resistance to crack initiation at the microscopic scale. Consequently, the micromechanical data acquired via nanoindentation provide critical localized inputs for calculating the surface energy (G) required for crack propagation and for evaluating the strain energy release rate. Furthermore, the mechanical non-homogeneity at the micro-nano scale revealed by this method is the fundamental mechanism for understanding why macroscopic cracks tend to propagate through weak matrices and how toughness is enhanced in composites through reinforcement phase design. By transcending the limitations of traditional macroscopic testing, nanoindentation has become an indispensable link in the cross-scale correlation of composition–microstructure–micromechanical properties–macroscopic damage resistance.

### 3.5. Cyclic Stress Fatigue Testing

The damage to refractories under cyclic thermal shock conditions is essentially a manifestation of stress fatigue behavior. During the test, the specimens are placed into a pre-heated electric furnace and rapidly heated to the target test temperature and held for a specific duration to ensure uniform heat distribution throughout the core of the material. Then the specimens are quickly removed from the furnace and immediately introduced to a cooling medium (water, flowing compressed air, or natural ambient air) for a set time to induce thermal stress. The test concludes either when the specimen physically fails (e.g., breaks into pieces or loses a specific percentage of its mass) or after a predetermined number of cycles are completed. Post-test mechanical strength may also be measured. By applying cyclic stress, the actual thermal shock environment can be effectively simulated, providing a more realistic reflection of the material’s damage resistance during rapid temperature fluctuations. Compared to traditional methods with limited cycles, such as water quenching or single mechanical tests, cyclic stress fatigue testing enables a more accurate characterization of damage resistance and service reliability under long-term thermal shock by establishing the relationship between the number of cycles and damage accumulation [8]. Li et al. [61] utilized the cyclic stress fatigue method to investigate the high-temperature fatigue behavior of 316L stainless steel welded joints. They found that tensile hold times reduce fatigue life and alter crack propagation modes, revealing the correlation mechanism between dislocation structures and cyclic hardening/softening. Zhang et al. [62] systematically studied the life characteristics and fracture mechanism transitions of GH4169 alloy under different phases and strain amplitudes through thermo-mechanical fatigue experiments, successfully simulating its cyclic deformation behavior based on the Chaboche viscoplastic model. Andreev et al. [63] employed strain-controlled cyclic fatigue testing to simulate the periodic strain loads during thermal shock, determining the strain limit and damage evolution of silica refractories under cyclic stress via three-point bending tests. The results indicated that at the same strain amplitude, the number of cycles to failure for crystallized fused silica was significantly higher than that of traditional silica bricks. The consistency between mechanical and thermal cycling trends confirms its superior life potential under actual thermal cycling conditions. This superiority stems from the fact that crystallized fused silica can withstand larger strains without rapid failure, attributed to the micro-cracks within its microstructure which blunt the primary crack tips and increase fracture energy. Consequently, its TSR exceeds that of traditional silica bricks, characterized by longer fatigue life and slower damage accumulation. Fatigue data provide failure cycle counts and damage evolution laws over a broad range of amplitudes, offering a more comprehensive evaluation than the degradation patterns observed under a single thermal shock regime. This provides a novel perspective for the assessment of TSR.

### 3.6. Machine-Learning-Based Assisted Prediction

Machine Learning (ML) is a core domain of Artificial Intelligence that empowers computers to acquire new knowledge and skills by analyzing vast datasets, thereby continuously enhancing their own performance. Currently, ML is increasingly being integrated into the performance characterization of refractories. By analyzing massive amounts of material compositions, processing parameters, and performance data, ML can accurately predict the high-temperature behavior of refractories and, in turn, optimize formulations through inverse design. Regarding TSR, ML models can deeply mine critical factors influencing stability, offering optimized strategies for developing more durable refractories with extended service lives. ML-assisted prediction of TSR is an inevitable trend in the industry. Currently, several integrated ML methodologies have already been implemented to characterize this property. The integration of machine learning marks a shift toward the intelligent development and smart manufacturing of refractory materials.

**Figure 11:**
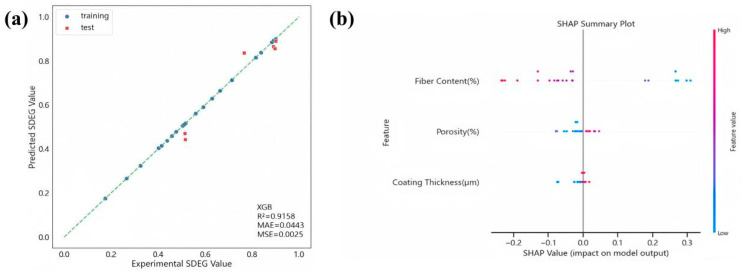
(**a**) Prediction results using XGBoost regression; (**b**) SHAP values of each feature for each sample based on XGBoost regression [64].

Sado et al. [16] conducted an in-depth study on the TSR of MgO-C refractories using machine learning methods based on Artificial Neural Networks (ANN). By constructing a three-layer back-propagation ANN model integrated with FEA simulation data, they successfully predicted key parameters such as steel shell temperature, maximum tensile stress, and maximum compressive stress. The model predictions showed high consistency with simulation results, with a temperature error of only 4 °C and stress errors around 4%. This demonstrates that ML can effectively optimize lining designs and enhance TSR, thereby extending service life and reducing maintenance costs. Deng et al. [64] evaluated the TSR of SiC-coated carbon fiber-reinforced carbon aerogel composites using a novel approach combining the Cohesive Finite Element Method (CFEM) and machine learning. CFEM was employed to simulate crack initiation and propagation in the coatings under thermal shock, creating a dataset used to train ML models, which significantly reduced reliance on traditional trial-and-error processes. The error in predicting residual stress ranged from 15.70% to 24.11%, while the developed ML model achieved a high coefficient of determination of 0.9171 when predicting the average stiffness degradation factor after three thermal shock cycles. Among various models, the XGBoost regression model exhibited superior predictive performance. As shown in Figure 11a, the predicted data points align closely with the 45-degree diagonal, indicating exceptional fitting accuracy. To interpret the model predictions, SHAP (Shapley Additive exPlanations) analysis was employed to identify key features and their contribution degrees, as shown in Figure 11b. Fiber content was found to have the most significant impact on TSR, which is consistent with simulation results; fibers enhance stability by improving internal toughness and stress distribution.

Despite the significant potential of machine learning in predicting the TSR of materials like MgO-C, the research and development of refractories generally face the challenges of “small sample sizes and high noise.” These issues often limit pure data-driven models in terms of reliability, generalization, and interpretability. To overcome this bottleneck, future research must shift from a sole reliance on data toward a path driven by the synergy of physical laws and data. Physics-Informed Neural Networks (PINNs) represent a pivotal breakthrough in this direction. The core concept is to integrate known physical laws—such as governing equations and boundary conditions—into the loss function of the neural network as soft constraints. By incorporating the R_Π_ theory based on the Buckingham Π theorem, Hasselman’s energy balance equations, or thermo-mechanical coupling equations as physical constraints, the model can be guided to learn solutions consistent with physical mechanisms even from limited and noisy experimental data. This approach not only reduces the dependency on massive, pristine datasets but also leverages physical priors to compensate for data deficiencies. Consequently, the predictions are grounded in physical principles rather than mere black-box extrapolation. Furthermore, such physics-embedded intelligent models can be deeply integrated with DIC and AE systems. This transition promises to shift the evaluation of refractory TSR from traditional post-hoc statistical prediction to physics-driven design and real-time intelligent maintenance, achieving a fundamental methodological transformation.

### 3.7. Conclusions

As demonstrated by the TSR evaluation methods discussed above, traditional thermal cycling tests, while intuitive, are inherently time-consuming and labor-intensive. In contrast, splitting tests provide a more targeted assessment of the material’s performance and fracture energy under tensile states. UPV and nanoindentation provide critical data from the perspectives of macroscopic non-destructive testing and microscopic mechanical properties, respectively, while cyclic stress fatigue testing simulates the actual performance of materials under real-world thermal shock conditions. Meanwhile, non-destructive dynamic monitoring techniques, represented by DIC and AE, enable the in-situ and real-time observation of crack initiation and propagation. In recent years, the introduction of ML has further expanded the research horizons, offering new possibilities for predicting performance and optimizing material designs from complex datasets. A comprehensive comparison of these evaluation methods is summarized in Table 5.

## 4. Conclusions and Prospect

Evaluating the thermal shock resistance (TSR) of refractories is a core component of lifecycle management, directly dictating equipment safety and service life in demanding environments like green metallurgy. Theoretical frameworks have advanced from Kingery’s thermo-elastic theory, which applies mainly to ideal dense materials, to Hasselman’s energy-based damage theory, which better explains crack dynamics in porous and low-strength refractories. While the emerging RΠ theory offers a promising novel approach, a unified theoretical framework capable of accurately modeling the entire process, from initial micro-cracking to catastrophic failure, remains a major challenge. Traditional thermal cycling tests are intuitive but labor-intensive and limited in scope. In contrast, splitting tests provide distinct advantages by accurately assessing tensile properties and pre-cracked steady-state fracture energy. The integration of non-destructive, in-situ monitoring techniques like Digital Image Correlation (DIC), Acoustic Emission (AE), and Ultrasonic Pulse Velocity (UPV) allows for the precise, real-time capture of crack evolution and energy dissipation mechanisms. Integrating Machine Learning (ML) with numerical simulations and material datasets is transitioning the field toward a data-driven paradigm, significantly enhancing R&D efficiency and predictive accuracy for material design.

Future evaluations of the TSR of refractories must focus on breaking through the limitations of current theoretical models to construct a cross-scale evaluation system that authentically reflects damage evolution under complex service environments. Priority should be given to investigating the influence of key parameters, such as characteristic crack length, brittleness parameters, and initial microcrack distributions, on damage patterns. This is essential to reconcile the discrepancies between thermo-elastic and energy-based theories, ultimately forming a unified theoretical framework capable of describing the entire process from crack initiation and propagation to catastrophic instability. Furthermore, concerted efforts are required to study the TSR of various refractories based on the R_Π_ theory, thereby establishing a novel framework for R_Π_ evaluation. It is crucial to identify the specific functional relationships and damage laws governing physical parameters, such as thermal conductivity and elastic modulus, in relation to TSR. These advancements will propel refractory evaluation methodologies toward a future characterized by standardization, cross-scale integration, and digitalization.

While evaluation methods have diversified, many advanced techniques remain confined to laboratory or “non-standard” applications. This results in poor data comparability across different studies, creating a significant R&D gap. Future efforts must focus on integrating non-destructive and in-situ monitoring techniques—such as wedge splitting, Digital Image Correlation (DIC), and Acoustic Emission (AE)—into international (ISO, ASTM) and national (GB) standard systems. The standardization of the wedge splitting method will be pivotal, as it enables the direct acquisition of key parameters for Hasselman’s energy-based damage theory and Harmuth’s brittleness evaluation theory, thereby facilitating the practical application of these theories in material design. Simultaneously, normalizing the operational procedures for dynamic monitoring technologies like DIC and AE will allow for the quantitative comparison of damage processes. This will provide the industry with reliable, unified criteria for crack initiation and propagation, effectively ending the reliance on highly empirical methodologies, such as traditional water quenching, and significantly elevating the overall R&D efficiency and precision of the industry.

The macroscopic thermal shock failure of refractories originates from the initiation and cross-scale evolution of microscopic defects. However, current evaluation methods are largely confined to a single scale, lacking an effective mechanism for cross-scale correlation. Consequently, future research must construct a Micro-Meso-Macro multiscale evaluation system. Specifically, nanoindentation can be utilized to characterize critical parameters, such as hardness, elastic modulus, and fracture toughness of the matrix, grain boundaries, and interfaces at the microscopic scale. This approach quantitatively reveals the impact of micro-defects on macroscopic fracture energy and thermal shock life, ultimately establishing predictive models that link composition and microstructure to service life, thereby enabling the tuning of macroscopic performance through microscopic design.

In the future of green metallurgy and intelligent manufacturing, the evaluation of refractories must undergo deep digitalization. An online damage warning system based on the Internet of Things (IoT) and smart technologies should be developed, with sensors embedded in high-temperature linings to collect real-time temperature and acoustic emission (AE) signals. These data are then fed into a digital twin, where intelligent models simulate the stress field and analyze AE features to identify early-stage damage. This enables thermal shock early warning and life prediction, driving a paradigm shift in refractory management from scheduled replacement to predictive maintenance, significantly enhancing both equipment safety and economic efficiency.

## Figures and Tables

**Table 1:** Comprehensive comparison of factors in Kingery’s thermoelastic theory.

Symbol	Core Criterion	Applicable Scenarios & Material	Limitations
R	Based on the critical temperature difference (ΔT_max_); failure occurs when thermal stress exceeds tensile strength.	Rapid heating/cooling (high heat transfer coefficient); Dense, flawless ideal ceramics.	Based on the assumptions of homogeneous and defect-free elastic mechanics, this model completely ignores the inherent pores and microcracks in refractories, leading to a failure in predicting the behavior of porous materials
R′	Incorporates thermal conductivity (λ); considers the material’s ability to dissipate heat	Slow heating/cooling (low temperature gradients); Dense, flawless ideal ceramics.	
R″	Incorporates thermal diffusivity (α) to characterize the ability to equalize internal temperature	Scenarios involving unsteady-state heat conduction and temperature homogenization; Dense ideal ceramics.	

**Table 2:** Comprehensive comparison of factors in Hasselman’s thermal shock damage theory.

Symbol	Core Criterion	Applicable Scenarios & Material	Limitations
$R^{\prime \prime \prime }$	Using the balance between elastic strain energy (W) and fracture energy (G) as the criterion, the crack propagation resistance of materials with the same fracture surface energy was compared	Applicable to scenarios involving initial short cracks; specifically designed for low-strength, porous refractory materials containing micro-cracks	Focusing solely on elastic strain energy, this approach is primarily used to compare materials with identical fracture surface energies, particularly when crack lengths vary and material properties are temperature-dependent
$R^{\prime \prime \prime \prime }$	By incorporating the fracture surface energy (G) into the R‴ parameter, the resistance to crack propagation for materials with varying fracture surface energies can be compared	Applicable to scenarios involving initial short cracks; specifically designed for low-strength, porous refractory materials containing micro-cracks	Accounting for both elastic strain energy and fracture surface energy, this approach enables the comparison of materials with diverse fracture surface energies, while considering varying crack lengths and temperature-dependent properties
$R_{st}$	Evaluating the resistance of materials to stable growth of pre-existing long cracks	Applicable to scenarios involving pre-existing long cracks; specifically designed for porous, micro-cracked, and low-strength refractory materials	The theoretical model still requires refinement, as it struggles to provide a unified description of the varying initial crack lengths found in real-world materials
$R_{st}^{\prime }$	Evaluating crack propagation based on R_st_ while incorporating thermal conductivity (λ) to assess materials with initial long cracks under severe thermal shock conditions	Applicable to scenarios involving initial long cracks and severe thermal shock conditions; specifically designed for porous, micro-cracked, and low-strength refractory materials	Similar to R_st_, the model provides insufficient consideration for the stochastic distribution of crack lengths and the temperature-dependency of material properties

**Table 3:** Calculated Π and R_Π_ of each sample [15].

Sample Number	Π_1_	Π_2_	Π_3_	R_Π_
S38	0.75 × 10^−3^	0.12 × 10^−2^	1.64	2.51
S45	0.57 × 10^−3^	0.15 × 10^−2^	0.84	1.56
S55	0.51 × 10^−3^	0.11 × 10^−2^	0.79	1.41
S65	0.43 × 10^−3^	0.11 × 10^−2^	0.58	1.12
L38	0.43 × 10^−3^	0.09 × 10^−2^	1.25	1.77
L45	0.56 × 10^−3^	0.12 × 10^−2^	0.80	1.48
L55	0.43 × 10^−3^	0.09 × 10^−2^	0.75	1.27
L65	0.36 × 10^−3^	0.09 × 10^−2^	0.57	1.02

**Table 4:** The TSR parameters and test results of different refractory materials.

Refractory	Value of TSR Factor	Thermal Cycling Test (Residual Strength Retention Rate)	Wedge Splitting Test (Fracture Energy)
Al_2_O_3_-C	8.6 K·m^1/2^ (R_st_) (w(Al_2_O_3_) = 66%)	71.7–79.4% (Thermal shock 5 times, air cooling)	370–439 J/m^2^ (low-carbon content)
MgO-C	8−9 MPa^−1^ (R‴) (w(MgO) > 96%)	47.0–87.3% (Thermal shock 1 time, water cooling)	283.1–374.1 J/m^2^ (w(MgO) > 75.75%)
MgO-Al_2_O_3_	0.30–2.35 mm (R′′′′) (w(MgAl_2_O_4_) < 30%)	52.0–78.3% (Thermal shock 3 times, air cooling)	196–326 J/m^2^ (w(MgAl_2_O_4_) < 34%)

**Table 5:** Comprehensive Comparison Table of Evaluation Methods.

Evaluation Method	Core Indicators	Theoretical Relevance	Industrial Application Suggestions
Thermal Cycling	Residual strength retention rate, number of cycles to failure	Based on the Critical Temperature Difference (ΔT_c_); correlates with Kingery’s thermoelastic theory	The most commonly used method for evaluating TSR
Brazilian Splitting Test	Tensile strength, crack evolution	Based on linear elastic assumptions; indirectly measures tensile strength via compressive loading	Widely applicable for evaluating tensile strength in engineering materials
Wedge Splitting Test	Specific fracture energy, characteristic length	Directly corresponds to Hasselman’s energy damage theory and Harmuth’s brittleness evaluation theory	Facilitates the observation of stable crack propagation from pre-existing notches
Ultrasonic Pulse Velocity	Ultrasonic wave velocity, velocity attenuation rate	Acoustic velocity attenuation reflects the evolution of internal damage factors.	Suitable for in-line quality inspection and internal damage assessment on production lines
Digital Image Correlation	Displacement field, strain field	Full-field, non-contact measurement for in-situ capturing of the FPZ evolution	Visual monitoring of dynamic crack evolution and strain localization
Acoustic Emission	AE hit/event count, energy, frequency	Captures elastic wave signals released by damage to reflect the cumulative internal damage process	Real-time monitoring of damage initiation and propagation under thermal shock
Nanoindentation	Hardness, elastic modulus, fracture work	Characterizes mechanical properties at the micro/nano-scale; correlates with micromechanical behavior	Used to reveal micro-strengthening mechanisms and phase-specific properties
Cyclic Stress Fatigue Test	Fatigue life, strain limits	Based on stress fatigue theory; simulates actual cyclic thermal shock service conditions	Realistically assesses the service reliability of materials under long-term thermal cycling
Machine Learning Assisted Prediction	Prediction accuracy, key parameter identification	Establishes complex non-linear mappings between “Composition–process–performance” via data mining	Enables efficient performance prediction and inverse design of materials

## Data Availability

No new data were created or analyzed in this study. Data sharing is not applicable to this article.
